# First person – Essi Wallén

**DOI:** 10.1242/dmm.052503

**Published:** 2025-06-18

## Abstract

First Person is a series of interviews with the first authors of a selection of papers published in Disease Models & Mechanisms, helping researchers promote themselves alongside their papers. Essi Wallén is first author on ‘ Effects of alcohol on the transcriptome, methylome and metabolome of *in vitro* gastrulating human embryonic cells’, published in DMM. Essi is a PhD researcher in the lab of Nina Kaminen-Ahola at Environmental Epigenetics Laboratory, Department of Medical and Clinical Genetics, Medicum, Faculty of Medicine, University of Helsinki, Helsinki, Finland, investigating how alcohol influences the early human development.


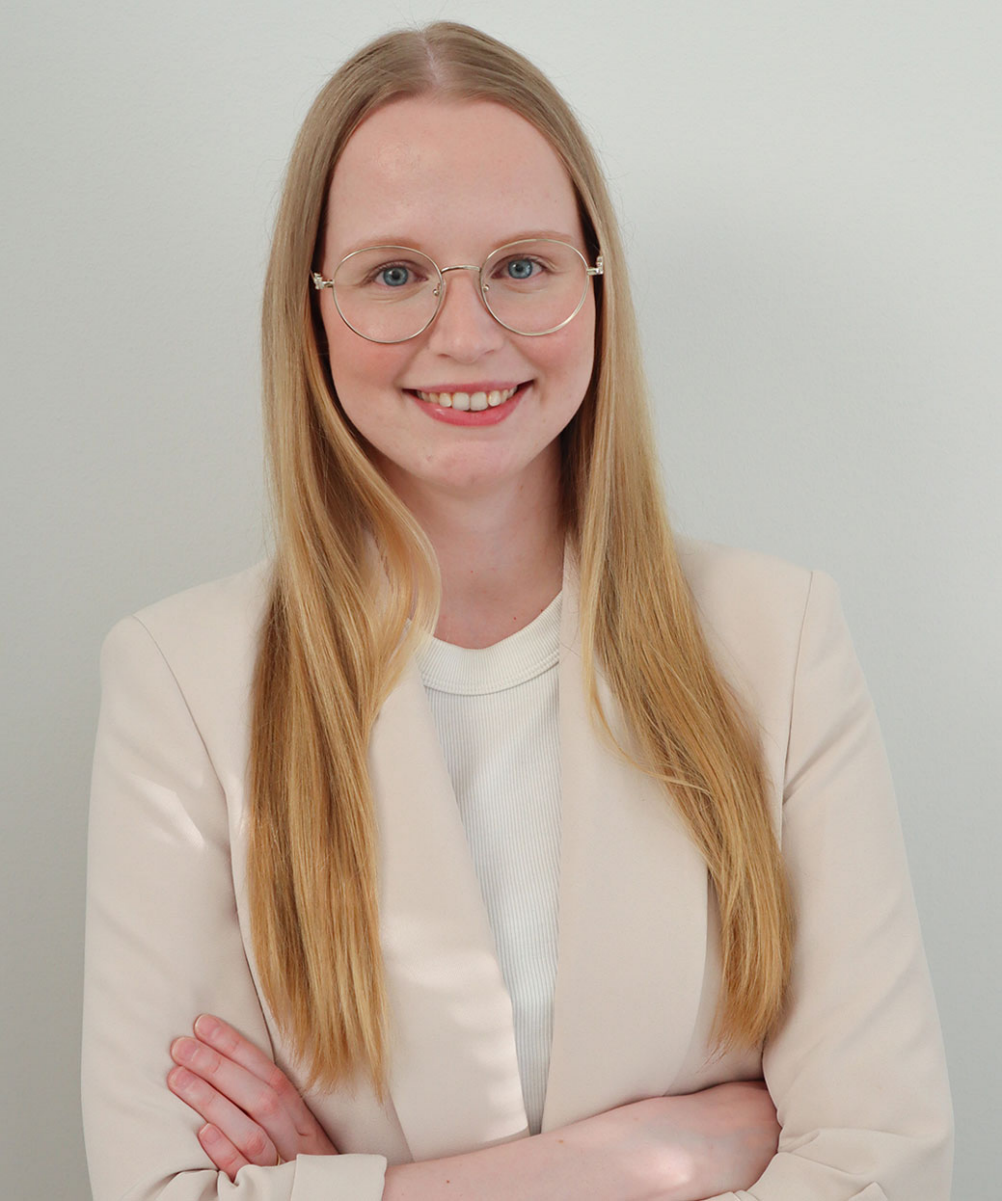


**Essi Wallén**

**Who or what inspired you to become a scientist?**

I feel fortunate to have grown up in an environment that nurtured my natural interest in science, particularly biology. I have always been fascinated by the mechanisms behind body functions and dysfunctions, and this curiosity often led me to ask my parents and biology teachers: “But why?”. As I learned more, I realized that these questions remain unanswered, and it inspired me to become a scientist, to seek answers to these questions. By uncovering answers and finding solutions, I also have the opportunity to help people in vulnerable positions, which further motivates me to be a scientist. Luckily, I have always received encouragement and support from my family and teachers to pursue a career as a scientist, and it has been truly valuable.

**What is the main question or challenge in disease biology you are addressing in this paper? How did you go about investigating your question or challenge?**

The first weeks of pregnancy are sensitive to environmental factors, and even small changes in developmental programming can lead to long-term consequences. Gastrulation, the process during which the three embryonic germ layers form, is considered one of the most critical events in development. However, due to ethical and technical limitations, our understanding of the mechanisms underlying prenatal alcohol exposure (PAE) and its early effects on development remains limited. It is still unclear whether the changes caused by early PAE may influence the characteristics of fetal alcohol spectrum disorders (FASD), an umbrella term for all developmental defects caused by PAE.

To tackle these limitations and to investigate the impact of alcohol on human germ layer cells, we utilized an *in vitro* model with subchronic moderate and severe alcohol exposures during the differentiation of human embryonic stem cells (hESCs) into germ layer cells. We exposed the cells to alcohol from the blastocyst-stage hESCs through to the differentiation into germ layer cells.

Given that gastrulation is a tightly regulated developmental process during which epigenetic reprogramming occurs, we were particularly interested in epigenetic gene regulation. We analyzed genome-wide gene expression by mRNA-sequencing and DNA methylation by EPIC Illumina microarrays. To further explore the underlying mechanisms, we performed metabolomic analysis by using non-targeted LC-MS method to examine changes in extracellular metabolites, aiming to understand the interplay between methylome, transcriptome, and metabolome.

**How would you explain the main findings of your paper to non-scientific family and friends?**

Alcohol exposure during pregnancy can have a broad impact on embryonic development, but the underlying mechanisms are still poorly understood. Studying the early weeks of pregnancy in humans is challenging, so we used a simple cell model to study this critical stage of development. We differentiated hESCs into three germ layers while simultaneously exposing them to alcohol. The formation of these germ layers is called gastrulation, and all human organs and tissues develop from these germ layers. Gastrulation occurs in the fifth week of pregnancy when women do not always know they are pregnant.

We found that severe alcohol exposure caused more changes than moderate levels of exposure, revealing a dose-response relationship between alcohol exposure and gene expression as well as metabolism. The most prominent changes were observed in ectodermal cells, which give rise to the nervous system and brain. PAE is recognized as a major cause of neurodevelopmental disorders. Many of the important developmental genes altered in the study have previously been linked to PAE and its associated features, such as heart and brain developmental disorders. However, further research is needed to determine how well this model and the alcohol exposures investigated by us correspond to actual exposure in humans.

**What are the potential implications of these results for disease biology and the possible impact on patients?**

PAE is one of the most significant causes of developmental disabilities worldwide. The findings of our current study contribute to a broader understanding of how alcohol affects the first differentiating cells in human. The study also provides a foundation for future human cohort studies with confounding factors.

Gaining a deeper understanding of the mechanisms underlying alcohol exposure will enhance diagnostics, prevention and treatment options, and significantly benefit those affected by alcohol during pregnancy. From a prevention perspective, our study supports existing recommendations to already abstain from alcohol when planning a pregnancy.

**Figure:**
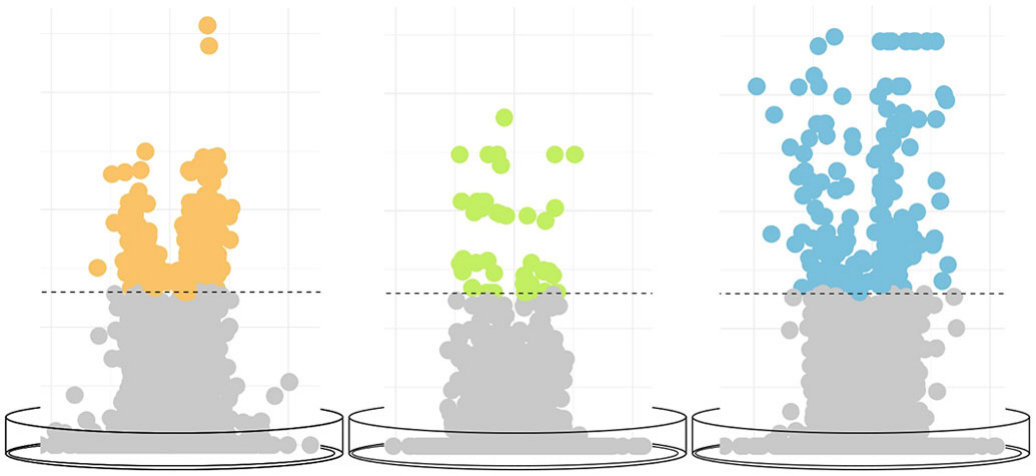
**We found the most prominent alcohol-induced DNA methylation changes in the *in vitro*-cultured ectodermal cells (blue) compared to the endodermal (yellow) and mesodermal cells (green).** Horizontal dashed lines indicate FDR<0.05.

**Why did you choose DMM for your paper?**

DMM is a respected open access biomedical journal publishing high-quality research; our research on human cell model examining the effects of environmental factor aligns well with its scope. We hope that the journal's diverse readership, which includes developmental biologists, toxicologists and clinicians, will find our work engaging. We also appreciate the solid review process. A noteworthy detail is DMM's commitment to sustainability: for every article published, the journal plants a tree.

**Given your current role, what challenges do you face and what changes could improve the professional lives of other scientists in this role?**

I see the biggest challenge for doctoral researchers as the uncertainty of research funding. The costs of research equipment and new analysis methods have been rising but research grants have not seen a comparable increase. The same situation applies to personal expenses, as many grants do not adequately cover living expenses. This uncertainty leads to increased stress and, unfortunately, drives many talented young researchers away from academia. It is essential to improve the availability of research funding and enhance transparency in the funding processes. Longer grant periods and adequate financial support would provide greater stability for researchers. Additionally, lengthening of publication times makes career advancement more challenging, as the quantity of research publications is a critical factor in the academic evaluation of researchers.

**What's next for you?**

I am continuing my PhD research with further analyses and manuscript preparation. The now published article focused on the effects of early PAE on embryonic cells. I am also investigating extra-embryonic cells, by using placental tissue from the early weeks of pregnancy as well as placental trophoblast organoids. Additionally, I am exploring in more detail how alcohol affects imprinted genes in various human cell types. These studies are part of a larger project for which our research group is studying how the prenatal environment affects the developmental programming and subsequent phenotype. In the long term, I aspire to continue my research career in academia, and secure a fulfilling postdoctoral position in developmental biology and epigenetics.

**Tell us something interesting about yourself that wouldn't be on your CV**

For me, the best way to relax is through cooking and baking. I enjoy planning new recipes, unleashing my creativity in the kitchen, and challenging my culinary skills with new ingredients and techniques. During my undergraduate studies, I organized popular vegan cooking nights for my fellow students, and I still love the opportunity to cook with friends and family. I believe that the best dishes often emerge from the chaos of a busy, slightly messy kitchen. Additionally, I love hiking and camping, as they perfectly combine my passions for the outdoors and great food. If there are not too many mosquitoes, fine dining can also be an option on a hiking trip.
